# ^18^F-DCFPyL PET/CT in advanced high-grade epithelial ovarian cancer: A prospective pilot study

**DOI:** 10.3389/fonc.2022.1025475

**Published:** 2022-10-13

**Authors:** Ur Metser, Roshini Kulanthaivelu, Tanya Chawla, Sarah Johnson, Lisa Avery, Douglas Hussey, Patrick Veit-Haibach, Marcus Bernardini, Liat Hogen

**Affiliations:** ^1^ Joint Department of Medical Imaging, University Health Network, Sinai Health Systems, Women’s College Hospital, University of Toronto, Toronto, ON, Canada; ^2^ Department of Biostatistics, University Health Network, Toronto, ON, Canada; ^3^ Division of Gynecologic Oncology, University Health Network, University of Toronto, Toronto, ON, Canada

**Keywords:** 18F-DCFPyL, PSMA, PET/CT, CT, ovarian cancer, diagnostic performance

## Abstract

**Objectives:**

Glutamate carboxypeptidase-II (GCP-II), a zinc metalloenzyme that resides in cell membrane, has been reported as overexpressed in the neovasculature of ovarian cancers. The study objective was to determine whether GCP-II targeted imaging with ^18^F-DCFPyL PET/CT can detect disease sites in women with advanced high-grade serous ovarian cancer (HGSOC).

**Materials and methods:**

Twenty treatment-naïve women with advanced HGSOC were recruited (median age 60 years). Prior to commencing therapy (primary cytoreductive surgery [n=9] or neoadjuvant chemotherapy [n=11]), subjects underwent routine staging with contrast-enhanced abdominopelvic CT (=CT), followed by ^18^F-DCFPyL PET/CT (=PET). CT and PET were reported independently using a standardized reporting template assessing 25 sites. The performance of PET was compared to CT in all subjects and to surgery and surgical histopathology in 9 patients who underwent primary cytoreductive surgery.

**Results:**

Of the 25 sites assessed in 20 patients, CT detected disease in 292/500 (58.4%) locations and PET detected disease in 171/500 (34.2%). Compared to CT the sensitivity (95% CI) of PET to detect disease in the upper abdomen, the gastrointestinal tract or the peritoneum was 0.29 (0.20,0.40), 0.21 (0.11,0.33) and 0.74 (0.64,0.82), respectively. In the surgical cohort, 220 sites in 9 patients were evaluated. The sensitivity and specificity of CT and PET were 0.85 versus 0.54 (p<0.001) and 0.73 versus 0.93 (p<0.001), respectively.

**Conclusion:**

Although ^18^F-DCFPyL has higher specificity than CT in detecting advanced HGSOC tumor sites, it detects less disease sites than CT, especially in the upper abdomen and along the gastrointestinal tract, likely limiting its clinical utility.

**Clinical trial registration:**

ClinicalTrials.gov, NCT03811899.

## Introduction

Epithelial ovarian cancer is the fifth most common cancer in women in the United States, and it has the highest fatality-to-case ratio of all the gynecologic malignancies (1). These tumors spread primarily by exfoliation of cells into the peritoneal cavity, but also by lymphatic and hematogenous dissemination. High-grade serous carcinomas (HGSOC) are the most common histologic subtype, with up to 75% of patients presenting with advanced-stage disease, for which surgery alone is not curative. Standard therapy consists of either primary cytoreductive surgery (PCS) followed by platinum-based chemotherapy or neoadjuvant chemotherapy (NACT) followed by interval cytoreductive surgery and further chemotherapy. Achieving complete resection in advanced ovarian cancer is often not feasible due to the multi-focal, disseminated nature of the disease (2). Numerous studies have shown that the degree of cytoreduction (i.e., the amount of residual disease at the completion of surgery) is directly correlated with survival. Patients with absence of gross residual disease after surgery have a much better outcome than those with optimal debulking (defined as residual sites of disease < 1 cm in diameter) or suboptimal debulking (defined as residual sites of disease ≥1 cm), with a 5-year survival estimated at only 15% after suboptimal debulking (2–5).

Accurate mapping of the distribution and volume of metastatic disease is vital for determination of the optimal therapeutic approach (PCS vs. NACT). Currently, most patients are staged with contrast-enhanced CT of the chest, abdomen, and pelvis (=CT); however, this tool has limited sensitivity and specificity, especially for disease in the mesentery or serosal surface of bowel (6–8). FDG PET/CT has been previously assessed with sensitivity and specificity of 78% and 68%, respectively, on a quadrant basis. Given the moderate performance measures, FDG PET has not been universally incorporated into the workup of these patients (9–11). A further non-invasive tool that would accurately map disease extent is needed to better select patients for primary therapy, reduce the rate of aborted surgery and associated morbidity, and hopefully improve patient outcomes.

Glutamate carboxypeptidase II (GCP-II) is a zinc metalloenzyme that resides in cell membranes, mostly on the extracellular side. It has various additional names including folate hydrolase and prostate specific membrane antigen (PSMA). It is expressed by normal tissues such as salivary and lacrimal glands, larynx, kidneys, bowel and prostate, as well as by multiple malignant tumors, often in the neovasculature of these tumors. GCP-II (=PSMA) has been extensively assessed in the setting of prostate cancer, especially in the setting of biochemical recurrence. In prostate cancer, PSMA PET has shown a very high sensitivity and moderately high specificity for the detection of recurrent or metastatic disease even when conventional imaging is negative (12–22). Initial report on the expression of GCP-II in neovasculature of gynecologic cancers including primary and metastatic epithelial ovarian cancer suggested high expression of GCP-II at immunohistochemistry in all 46 cases of ovarian cancer assessed (23). These findings were the impetus for the current study. The main aim of the current study was to determine whether GCP-II targeted imaging with ^18^F-DCFPyL PET/CT (=PET) can detect sites of disease in women with advanced HGSOC and to compare sites of disease detected on PET to CT and to intra-operative findings and surgical histopathology.

## Patients and methods

### Study design

This is an institutional ethics review board approved, single arm, prospective pilot study (ClinicalTrials.gov: NCT03811899). Written informed consent was obtained from all participants. The inclusion criteria were: 1. Age ≥18 years; 2. Cytological or histological diagnosis of high grade epithelial ovarian cancer; 3. Clinical stage III or IV, under consideration for PCS or NACT; 4. Contrast-enhanced CT abdomen and pelvis within 6 weeks of PET. Exclusion criteria included: 1. Evidence of epithelial ovarian cancer of the following histological subtypes: mucinous, low grade serous, low grade endometrioid and low-malignant potential tumors or metastases from other primary tumor; 2. Inability to complete study procedures (contraindication for PET as per institutional guidelines such as pregnancy, or participant’s inability to lie still for 30 minutes). Demographic and clinical data include age, FIGO (International Federation of Gynecology and Obstetrics) stage, serum CA-125 at presentation, and surgical outcomes were tabulated.

### Study procedures

#### ^18^F-DCFPyL PET/CT

PET was performed 90-120 minutes (mean ± SD: 100.3 ± 9.7) after injection of 310 ( ± 16.8) MBq of ^18^F-DCFPyL. During uptake time, water soluble oral contrast was given for bowel opacification on CT. Patients were positioned supine on the imaging couch with arms outside of the region of interest. Images were obtained from the skull base to the upper thighs. PET was performed on a Biograph mCT 40 scanner (Siemens Healthcare, Erlangen, Germany). Low dose CT without intravenous contrast was used for attenuation correction as per standard departmental protocols. Overall, 5-9 bed positions were obtained as per patient height (2-5 min/bed position).

#### CT protocol

The contrast-enhanced CT scan were performed by using the Aquilion 64 or Aquilion ONE CT (Canon Medical Systems). The scanning parameters were: tube voltage 120 kV and tube determined using automatic exposure control (^SURE^exposure). For Aquilon ONE scan parameters were as follows: 1–3 mm slice thickness; 2.4 mm slice interval; helical pitch = 65; pitch factor =0.813. For Aquilon 64 scan parameters were as follows: 1-5mm slice thickness, 2.5 mm slice interval; helical pitch: 53; pitch factor= 0.828. Images were obtained after intravenous administration of 100 ml of 300 mg of iodine per milliliter of nonionic contrast material (Ultravist 370; Schering) using a power injector through an 18-gauge at a rate of 3 ml/s. Coronal and sagittal reformats of the dataset were also obtained.

#### Imaging interpretation & reporting template

CT and PET imaging data sets were interpreted independently. When present, primary tumor, nodal, peritoneal and visceral metastases on CT were recorded by one of 2 readers (TC, SJ; with 21 and 9 years of experience) using standard diagnostic criteria (24). PET was interpreted in consensus by 2 readers (UM, RK with 20 and 5 years of experience). In general, on PET, focal tracer accumulation greater than background activity, which could not be attributed to physiological activity, or a benign entity were recorded. SUVmax at all tumor sites and PSMA score relative to reference tissues, as previously described, were documented (25). All disease sites on either modality were tabulated using a standardized synoptic reporting template evaluating 25 stations in the abdomen and pelvis (Appendix A). These included assessment of: 1. Primary ovarian tumor/s; 2. Nodal metastases (below and/or above the renal veins); 3. Peritoneum (8 stations); 4. Gastrointestinal tract (5 stations); 5. Upper abdomen (9 stations).

#### Reference standard

A head-to-head comparison of lesion detection on CT and PET was performed for all patients. For the subset of patients who underwent primary cytoreductive surgery findings on CT and PET were compared to intra-operative findings and surgical histopathology using the same synoptic reporting template. The detection rate, sensitivity, specificity, positive predictive value, negative predictive value, and overall accuracy were calculated for each modality according to the standard of reference for all evaluable stations.

### Statistical analysis

Sensitivity and specificity of PET were calculated on a per-lesion basis against standard of care contrast-enhanced CT. Calculations were performed across all lesions as well as at the local and regional level with exact confidence intervals as per Collett (26). A per-lesion analysis assumes lesions within patients are independent. To adjust for potential similarities of the assessment of lesions within patients a second analysis of the sensitivity and specificity was undertaken using generalised estimating equations (GEE) and an exchangeable working correlation structure as in Smith and Hadgu (26).

A sub-sample of nine patients underwent PCS with surgical histopathology. Sensitivity and specificity of both CT and PET were evaluated against the surgical standard for this subsample. Exact binomial tests (27, 28) were used to test the null hypothesis that the sensitivity and specificity of the tests was equal across regions, but the subsample was too small to detect differences at the lesion level.

## Results

**Figure 1:**
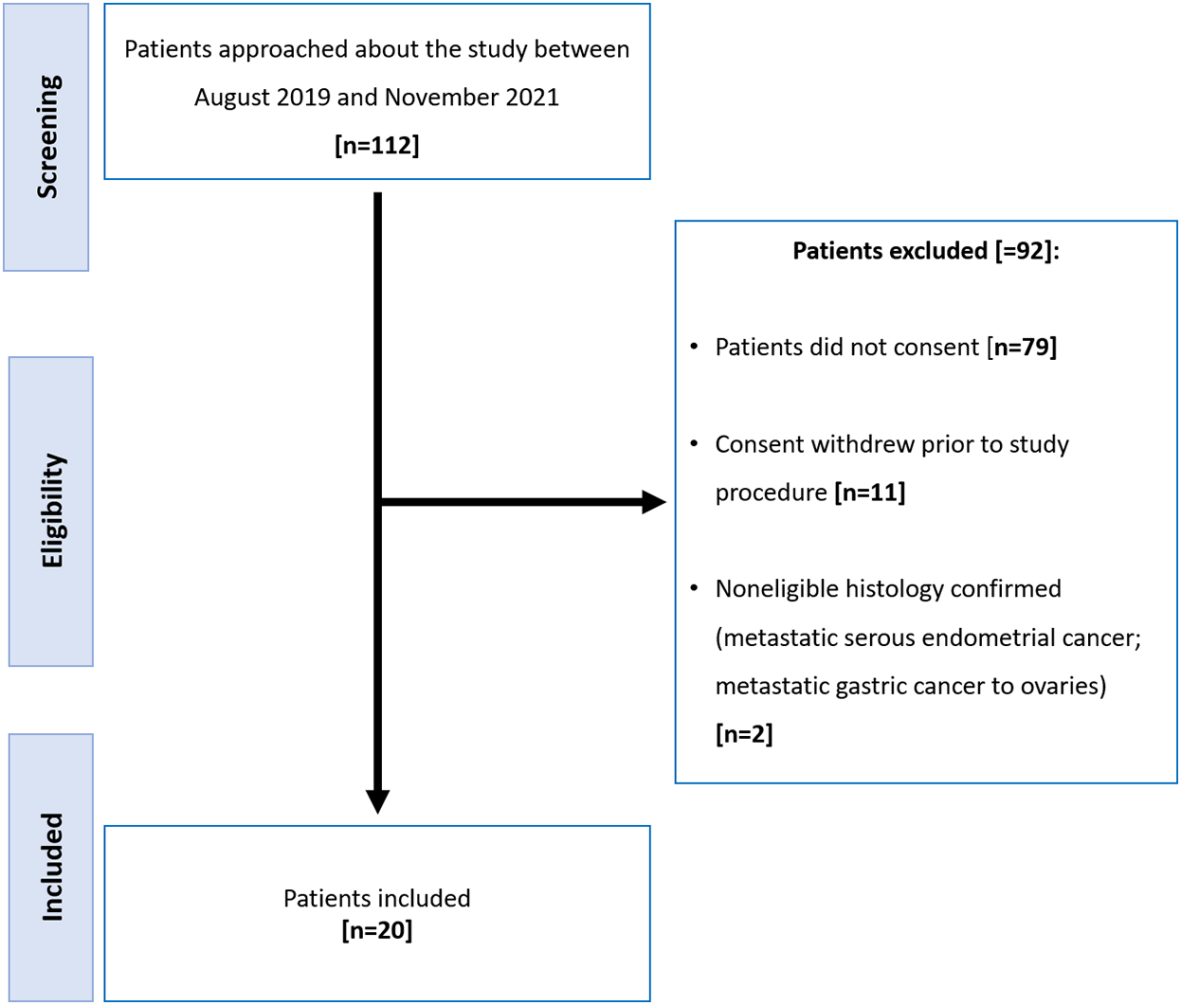
Patient flowchart.

Of the 112 participants approached to participate, 92 were excluded (**Figure 1**). There were 20 women (median age, 60 years; range: 38-83) with histologically proven high grade epithelial ovarian cancer included of whom 11 had NACT and 9 underwent PCS. Clinical data including stage, serum CA-125 and surgical outcome are summarized in **Table 1**.

**Table 1:** Summary of clinical parameters.

		Number of participants=
**Stage**	IIIC	13
	IV	7
**Serum CA-125 (U/ml)**	Mean ± SD	2238± 4100
	Range (min., max)	(64, 19154)
**Surgical Outcome**	Complete cytoreduction	8
	Optimal debulking (< 1cm)	10
	Suboptimal debulking	2

### Detection rate on PET

Of the 500 stations assessed in all 20 patients, CT detected disease in 292/500 (58.4%) and PET detected disease in 171/500 (34.2%). ^18^F-DCFPyL uptake when visible at disease sites was generally low or low to moderate with a mean SUVmax (± SD) of 4.2 ± 1.9 (range: 1.2-10.9). Of all 171 lesions assessed on PET, PSMA scores were 0, 1, and 2 in 8/171 (4.7%), 130/171 (76%), 33 (19.3%), respectively. No lesion with PSMA score of 3 was recorded.

Primary tumors were detected in 19/20 participants (95%) on both PET and CT. The performance measures of PET compared to CT for detection of disease for all evaluated stations and for metastatic sites grouped by anatomical location are presented in **Table 2** including the GEE adjusted measures, correcting for lack of independency of multiple lesions within the same patient.

**Table 2:** Performance measures of PET with CT as the reference for all stations assessed, and for the various stations grouped by anatomic location (excluding primary tumors).

STATISTIC		ALL STATIONS(n=500)	LYMPH NODES(n=40)	UPPER ABDOMEN(n=180)	GI TRACT(n=100)	PERITONEUM(n=160)
		*Value(95% CI)*				
**Prevalence**		0.58 (0.54, 0.63)	0.40 (0.25, 0.57)	0.51 (0.44, 0.59)	0.63 (0.53, 0.72)	0.64 (0.56, 0.71)
	***GEE adjusted***	0.58	0.40	0.51	0.63	0.64
**Sensitivity**		0.49 (0.43, 0.55)	0.56 (0.30, 0.80)	0.29 (0.20, 0.40)	0.21 (0.11, 0.33)	0.74 (0.64, 0.82)
	***GEE adjusted***	0.49 (0.42, 0.56)	0.44 (0.21, 0.66)	0.30 (0.18, 0.42)	0.21 (0.10, 0.31)	0.74 (0.62, 0.86)
**Specificity**		0.87 (0.81, 0.91)	0.79 (0.58, 0.93)	0.93 (0.86, 0.97)	0.97 (0.86, 1.00)	0.72 (0.59, 0.83)
	***GEE adjusted***	0.86 (0.80, 0.93)	0.72 (0.53, 0.91)	0.93 (0.88, 0.99)	0.97 (0.93, 1.02)	0.73 (0.60, 0.86)
**PPV**		0.84 (0.77, 0.89)	0.64 (0.35, 0.87)	0.82 (0.65, 0.93)	0.93 (0.66, 1.00)	0.82 (0.73, 0.90)
**NPV**		0.55 (0.49, 0.60)	0.73 (0.52, 0.88)	0.56 (0.47, 0.64)	0.42 (0.31, 0.53)	0.61 (0.48, 0.72)
**Accuracy**		0.65 (0.60, 0.69)	0.70 (0.53, 0.83)	0.61 (0.53, 0.68)	0.49 (0.39, 0.59)	0.73 (0.66, 0.80)

GI, gastrointestinal; CI, confidence interval; PPV, positive predictive value; NPV, negative predictive value. GEE, generalised estimating equations.

### Performance of CT and PET with surgery as reference standard

**Figure 2:**
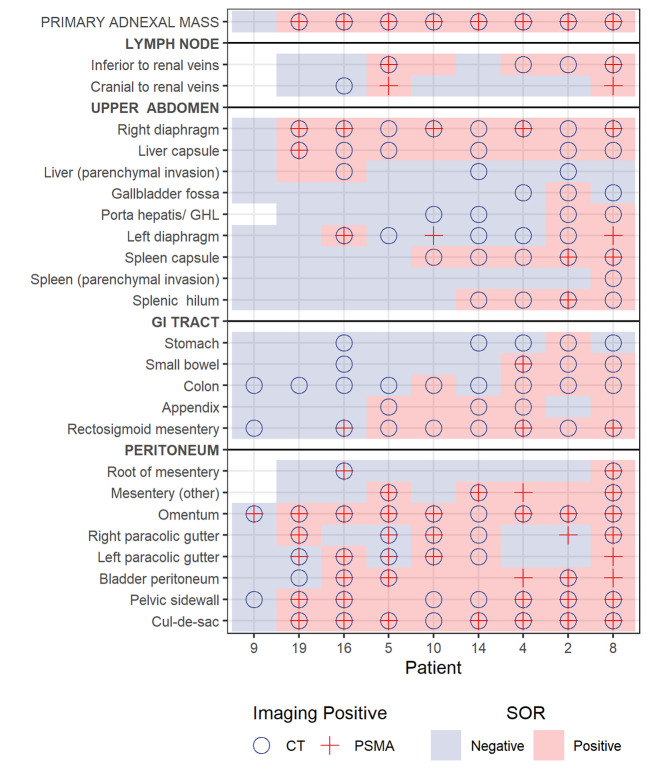
Plot showing lesion detection by site for each of the 9 patients who underwent primary cytoreductive surgery. Blue background and pink background denote negative or positive station according to reference standard, respectively. A circle notes positive on CT and cross notes positive on PET.

Nine participants underwent PCS with 220 evaluable stations (data were missing for 5 stations in one patient). There were 54 stations that were positive on CT, PET and at surgery; and 76 stations that were negative on CT, PET and at surgery. The overall performance measures of CT, and PET with surgery and surgical histopathology as the reference standard are presented in **Table 3**. Disease detection on imaging in the various stations evaluated with comparison to surgery as the reference standard is depicted in **Figure 2**; the sensitivity and specificity of CT and PET in identifying metastatic sites compared to surgery and surgical histopathology are presented in **Table 4**.

**Table 3:** Performance measures of CT, and PET with surgery and surgical histopathology as the reference standard for all evaluated stations (n=220).

STATISTIC	CT *(95% CI)*	PET *(95% CI)*	*p-value*
**Sensitivity**	0.85 (0.77, 0.91)	0.54 (0.44, 0.63)	<0.001
**Specificity**	0.73 (0.63, 0.81)	0.93 (0.86, 0.97)	<0.001
**PPV**	0.77 (0.68, 0.84)	0.88 (0.78, 0.95)	
**NPV**	0.82 (0.73, 0.89)	0.66 (0.57, 0.73)	
**Accuracy**	0.79 (0.73, 0.84)	0.73 (0.66, 0.78)	

PPV, positive predictive value; NPV, negative predictive value; CI, confidence interval.

**Table 4:** Sensitivity and specificity of CT and PET in identifying metastatic sites compared to surgery.

	Sensitivity			Specificity		
	CT*(95% CI)*	PET*(95% CI)*	*p-value*	CT*(95% CI)*	PET*(95% CI)*	*p-value*
**LYMPH NODES**	0.57 (0.18, 0.90)	0.57 (0.18, 0.90)	1	0.89 (0.52, 1.00)	1.00 (0.66, 1.00)	1
**UPPER ABDOMEN**	0.88 (0.73, 0.97)	0.32 (0.17, 0.51)	**<0.001**	0.80 (0.66, 0.91)	0.98 (0.88, 1.00)	**0.021**
**GI TRACT**	0.89 (0.67, 0.99)	0.16 (0.03, 0.40)	**<0.001**	0.54 (0.33, 0.73)	0.96 (0.80, 1.00)	**<0.001**
**PERITONEUM**	0.82 (0.68, 0.92)	0.78 (0.63, 0.89)	0.75	0.72 (0.51, 0.88)	0.76 (0.55, 0.91)	1

GI, gastrointestinal; CI, confidence interval.

Bold values are those with statistical significance.

## Discussion

**Figure 3:**
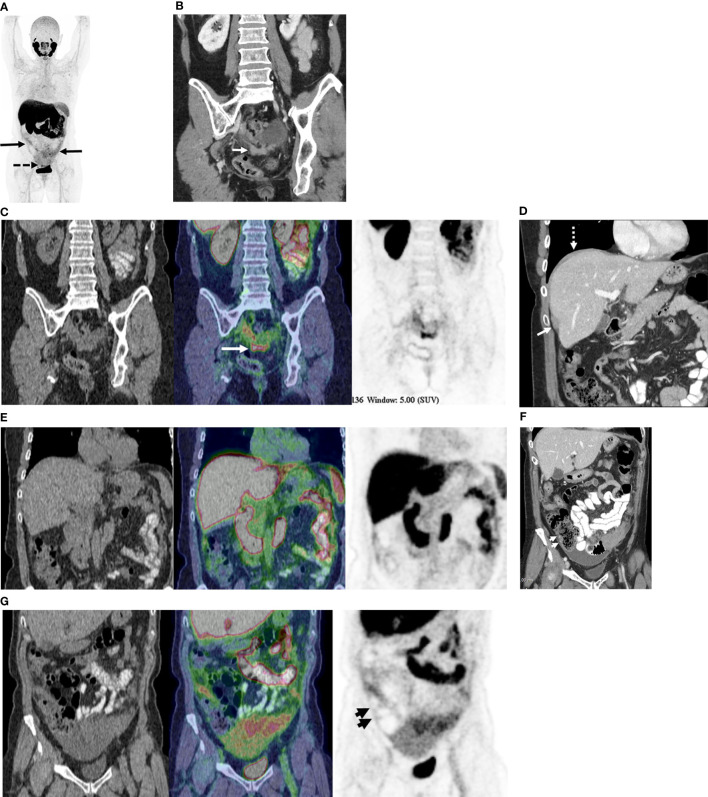
68-year old with stage IIIC high-grade serous ovarian cancer. **(A)** Maximum Intensity Projection image of 18F-DCFPyL shows mild to moderate radiotracer uptake in omental metastases (solid arrows) and moderate radiotracer uptake in pelvic peritoneal metastases (dotted arrows). **(B)** Coronal contrast enhanced CT image shows peritoneal deposits in the posterior cul-de-sac (arrow). **(C)** Coronal PET/CT image (CT - left, fused PET/CT image – middle; PET – right) corresponding to B shows moderately radiotracer uptake in same metastatic deposit (concordant CT and PET). Metastatic disease was confirmed at surgery. **(D)** Coronal contrast enhanced CT image shows metastatic disease on right diaphragm (dotted arrow) and along capsular surface of liver (solid arrow). **(E)** Coronal PET/CT image (CT - left, fused PET/CT image – middle; PET – right) corresponding to D show no focal radiotracer uptake visible on right diaphragm or liver capsule. Surgical pathology confirmed CT findings of metastatic disease at these sites. **(F)** Coronal contrast enhanced CT image shows focal thickening along right lateral wall of ascending colon (short arrows), suspected to represented serosal metastasis. **(G)** Coronal PET/CT image (CT - left, fused PET/CT image – middle; PET – right) corresponding to F shows no focal radiotracer uptake on the serosal surface of the ascending colon. No serosal disease on surface of the right colon was found at surgery.

In women with advanced HGSOC, ^18^F-DCFPyL (PSMA) PET/CT detects fewer metastatic sites of disease as compared to standard of care contrast-enhanced CT, but at a higher specificity. Although only a subsample of the study population underwent PCS, in these participants, surgery and surgical histopathology was used as the reference standard to compare the performance of CT and PET. Comparison of the sensitivity and specificity of modalities in this subsample is based on the assumption of independent lesions. The comparison of per lesion and GEE analyses indicates that this assumption is reasonable for most regions, with little difference between the adjusted and per lesion values. The performance of PET was especially poor for lesions in the upper abdomen and along the gastrointestinal tract. This is likely due to limited expression of GCP-II at tumor sites, as depicted with ^18^F-DCFPyL PET/CT, along with the high background activity in the liver and spleen and in segments of the gastrointestinal tract, limiting detection of subdiaphragmatic or capsular hepatic metastases and serosal deposits (**Figures 3A–G**). These findings suggest that GCP-II targeted imaging with ^18^F-DCFPyL PET/CT in women with advanced HGSOC is likely of limited clinical utility.

Preclinical studies on the expression of GCP-II in ovarian cancer have shown conflicting results. Wernicke et al. examined the expression of PSMA in neovasculature of gynecologic cancers including primary and metastatic ovarian cancer (23). The authors showed a high expression of PSMA at immunohistochemistry in all 46 cases of ovarian cancer assessed, a report which provided the impetus for the current study. A further, more recent study published by Aide et al. assessed 32 patients with 57 samples (including 25 samples obtained after chemotherapy). The authors demonstrated the quasi-absence of PSMA expression within serous epithelial ovarian cancers. Authors showed no correlation with resistance to chemotherapy and non-evolution of PSMA expression during the treatment course (29). Our findings are more in line with the results of Aide et al, with most disease sites in women with HGSOC showing low level ^18^F-DCFPyL (PSMA) uptake and the majority of lesions assigned a PSMA score of 1 (≥ blood pool activity and lower than liver uptake). Despite the ongoing debate on the optimal management and timing of surgery in women with advanced HGSOC and conflicting results in various trials (3, 30–32), accurate delineation of disease extent along with several other predictive parameters is crucial for personalizing management, with the goal of offering PCS to women in whom it is feasible to achieve cytoreduction to no gross residual disease (33). Although the results of the current study suggest that ^18^F-DCFPyL (PSMA) PET is not a promising modality for imaging of advanced high-grade ovarian cancers, we believe the study protocol developed including detailed comparison of disease mapped on imaging to findings at surgery and surgical histopathology can be utilized in future trials assessing other potential molecular probes targeting receptors or the tumor microenvironment in HGSOC.

Over 80% of patients with advanced HGSOC will experience recurrence within 59 months from initial treatment, with median progression free survival of ~14-15 months (31, 32). One of the proposed mechanisms for the high recurrence rates, is the development of drug resistance to platinum-based chemotherapy, including in patients who were initially responsive to platinum-based chemotherapy protocols. This disease course encourages exploration of new adjuvant therapies to improve disease control and improves outcomes. One example could be utilization of radionuclide therapy in a theranostic approach, where a specific biomarker is employed to image and to deliver targeted radiotherapy selectively to tumor sites. This approach has been effective in a few malignancies including metastatic prostate cancer where the provision of ^177^Lu-PSMA-617 in men with advanced PSMA-avid metastatic castration-resistant prostate cancer has been shown to improve progression-free survival and overall survival compared to standard care (34). A prerequisite for success of a theranostic approach is high avidity of the biomarker at tumor sites. In addition to demonstrating limited utility of ^18^F-DCFPyL PET/CT as a diagnostic tool in the staging of women with HGSOC, our findings also suggest that further exploration of PSMA as a target for a theranostic approach is unlikely to be productive in women with advanced ovarian cancers.

In conclusion, although ^18^F-DCFPyL has higher specificity than CT in detecting advanced HGSOC tumor sites, it detects less disease sites than CT, especially in the upper abdomen and along the gastrointestinal tract, limiting its clinical utility as a diagnostic tool. Further imaging biomarkers with high target affinity are needed to improve disease detection and for a theranostic approach to be considered in women with advanced high-grade epithelial ovarian cancers.

## Data availability statement

The original contributions presented in the study are included in the article/**Supplementary Material**. Further inquiries can be directed to the corresponding author.

## Ethics statement

The studies involving human participants were reviewed and approved by UHN Research Ethics Board. The patients/participants provided their written informed consent to participate in this study.

## Author contributions

UM, LH: conceptualization, methodology, resources and supervision. RK, TC, SJ, LA, DH: investigation and formal analysis. PV-H, MB: writing, review and editing. All authors read and approved the final manuscript.

## Conflict of interest

The authors declare that the research was conducted in the absence of any commercial or financial relationships that could be construed as a potential conflict of interest.

## Publisher’s note

All claims expressed in this article are solely those of the authors and do not necessarily represent those of their affiliated organizations, or those of the publisher, the editors and the reviewers. Any product that may be evaluated in this article, or claim that may be made by its manufacturer, is not guaranteed or endorsed by the publisher.

## Abbreviations

Gcp-ii, Glutamate carboxypeptidase II; PCS, primary cytoreductive surgery; NACT, neoadjuvant chemotherapy.
