# Supplementary figures and images for: 18F-DCFPyL PET/CT in advanced high-grade epithelial ovarian cancer: A prospective pilot study
Source: Front Oncol. 2022 Oct 13;12:1025475. doi: 10.3389/fonc.2022.1025475 (PMC9606351; doi:10.3389/fonc.2022.1025475)

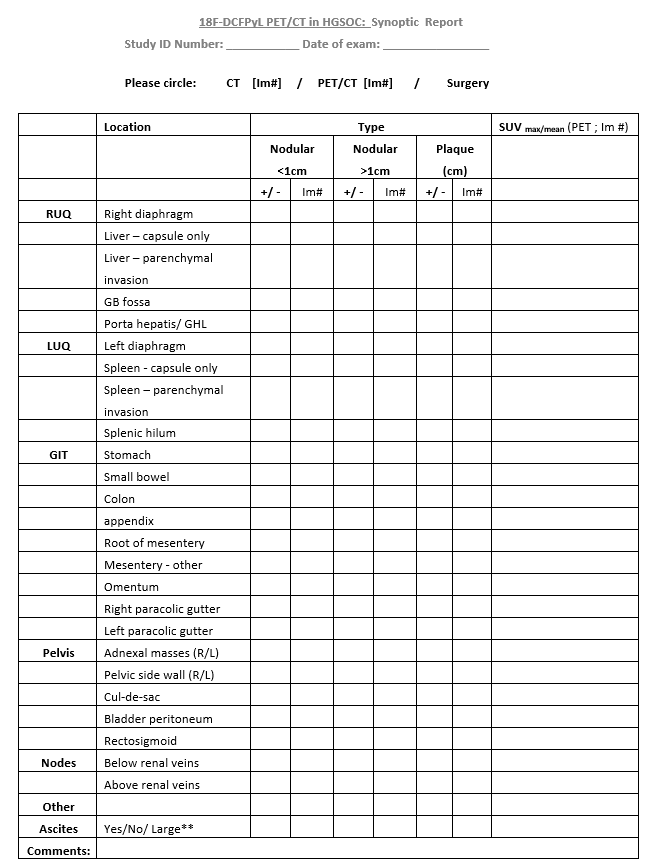

Supplement: Supplementary file 1 [file Image_1.tif]
